# Investigation of the Effect of Amniomax on Lidocaine-Induced Toxicity in Healthy Colon Cell Culture

**DOI:** 10.3390/biomedicines13051074

**Published:** 2025-04-29

**Authors:** Seçil Azime Karakuş, Ayten Saraçoğlu, Eray Metin Güler, Kübra Bozali, Ceren Önal, Yekbun Bulun, Tomasz Gaszyński, Paweł Ratajczyk, Kemal Tolga Saraçoğlu

**Affiliations:** 1 Department of Anesthesiology and Reanimation, University of Health Sciences Turkey, Başakşehir Çam and Sakura City Hospital, Istanbul 34480, Türkiye; usul.secil@gmail.com; 2 Department of Anaesthesiology, University of Florida College of Medicine, Jacksonville 8th St W Florida, Gainesville, FL 32209, USA; kemaltolgasaracoglu@gmail.com; 3 Department of Medical Biochemistry, Haydarpasa Numune Health Application and Research Center, Istanbul 34480, Türkiye; eraymetinguler@gmail.com; 4 Department of Medical Biochemistry, Hamidiye Faculty of Medicine, University of Health Sciences Turkey, Istanbul 34480, Türkiye; kubrabozalii@gmail.com; 5 Department of Medical Biochemistry, Hamidiye Institute of Health Sciences, University of Health Sciences Turkey, Istanbul 34480, Türkiye; 6 Department of Anesthesiology and Reanimation, Ağrı Research and Training Hospital, Ağrı 04200, Türkiye; cerenonl@gmail.com; 7 Department of Anesthesiology and Reanimation, Bingöl State Hospital, Bingöl 12000, Türkiye; dr.ybulunn@hotmail.com; 8 Department of Anesthesiology and Intensive Therapy, Medical University of Lodz, 90-153 Lodz, Poland; pawel.ratajczyk@umed.lodz.pl

**Keywords:** lidocaine, AmnioMax^®^, cytotoxicity, cell culture, viability, apoptosis

## Abstract

**Background:** Lidocaine (LIDO) toxicity is a critical concern in regional anesthesia, with no specific antidote currently available. While lipid emulsions are commonly used as rescue agents in cases of local anesthetic systemic toxicity (LAST), their efficacy is inconsistent, and their safety remains controversial. AmnioMax^®^ (AMX), a specialized cell culture medium enriched with growth factors and bioactive molecules, has the potential to offer cytoprotective effects. This study aims to investigate the therapeutic efficacy of AMX in mitigating lidocaine-induced cytotoxicity and to explore its protective mechanisms at the cellular level. **Methods:** Healthy colon cells (CCD-18Co) were used in this study. Four experimental groups were established as follows: control, LIDO, AMX, and LIDO + AMX. Cellular viability in the control group was set at 100%. LIDO was administered at concentrations ranging from 0.06 to 10%, AMX at 0.625–100%, and LIDO + AMX at 60% of the half-maximal effective concentration (EC_50_) combined with LIDO (0.06–10%). Cells were incubated for 24 h, after which cellular viability, DNA damage, apoptosis, intracellular reactive oxygen species (iROS), intracellular calcium (Ca), mitochondrial membrane potential (MMP), and glutathione (GSH) were evaluated. **Results**: LIDO exposure led to a concentration-dependent decrease in viability compared to the control group (*p* < 0.001), while AMX significantly increased viability (*p* < 0.001). In the LIDO + AMX group, viability was also reduced (*p* < 0.001); however, cytotoxicity was significantly lower than in the LIDO group (*p* < 0.05). Both the LIDO and LIDO + AMX groups showed increased iROS levels, DNA damage, and apoptosis (*p* < 0.001), along with the decreased MMP and GSH levels (*p* < 0.001) compared to the control. However, in the LIDO + AMX group, iROS, DNA damage, and apoptosis were significantly lower than in the LIDO group (*p* < 0.01), MMP levels were increased (*p* < 0.001), and no significant difference was observed in GSH levels. **Conclusions:** AMX demonstrated cytoprotective effects against LIDO-induced cytotoxicity, suggesting its potential as an alternative therapeutic agent for LAST.

## 1. Introduction

In recent years, the use of ultrasound-guided regional anaesthesia has become increasingly widespread. Despite advancements in imaging techniques, the risk of local anaesthetic systemic toxicity (LAST) continues to pose a life-threatening threat [1]. While the exact mechanisms of the neurotoxicity and cardiotoxicity that occur as lidocaine plasma levels rise are not fully elucidated, it is believed that local anaesthetic cytotoxicity is likely a major contributing factor [2].

Numerous animal studies have shown that lidocaine can cause apoptosis in cardiovascular disease by disrupting calcium ion balance, depending on the concentration and duration of exposure [3]. Furthermore, research has demonstrated that lidocaine increases apoptosis and decreases cell viability in both rat and human osteosarcoma cells in a concentration- and exposure-dependent manner [4].

Lipid emulsions, initially developed as parenteral nutrition preparations, are now included in LAST treatment protocols [5,6]. However, they are not specific antidotes for local anaesthetics. They are used as rescue agents in poisonings involving fat-soluble drugs such as antiarrhythmics, organophosphates, or psychotropic agents [7,8]. The use of lipid emulsions for LAST has been reported as “off-label” by the Food and Drug Administration [5]. The effectiveness of lipid emulsions can vary for different types of local anaesthetic agents because the pharmacokinetics and tissue distribution volume of a local anaesthetic agent can influence the efficacy of the lipid emulsion. Lipid particles are negatively charged. Therefore, the binding affinity of different local anaesthetics, which vary in positive charge, can also vary [9]. On the other hand, lipid emulsions can temporarily absorb local anaesthetics from heart and brain tissues. One study reported that lipid emulsion could only reduce the peak concentration of local anaesthetics by approximately 26% to 30% [10]. Due to these reasons, the reliability of using lipid emulsions has begun to be questioned. A systematic review analyzed the results of 114 studies, including 87 human and 27 animal studies, and reported that intravenous lipid emulsion administration led to serious mortal outcomes such as acute renal injury, acute lung injury, ventilation–perfusion mismatch, fat embolism, pancreatitis, venous thromboembolism, and even cardiac arrest [11].

For over a century, amniotic membranes and fluid have been used to naturally seal tissue defects and create a protective environment to prevent heat and fluid loss in open wounds. Nowadays, amnion treatments are commonly used in fields like ophthalmology, plastic surgery—especially for burns and wound healing—and certain orthopaedic conditions [12].

Amniotic fluid is a fluid that surrounds the developing foetus, providing essential nutrients for foetal growth while also serving a mechanical protection role [13]. One study has shown that amniotic fluid contains subpopulations of cells with stem cell properties capable of proliferation and differentiation. Based on these advancements, there is growing consideration that stem cells sourced from amniotic fluid could facilitate cellular regeneration at a fundamental level [14]. Although previous research highlights the therapeutic potential of AmnioMax^®^ (AMX), further studies are necessary to evaluate its routine application in LAST treatment. Our study provides additional evidence supporting the cytoprotective role of AMX. It is well established that LIDO cytotoxicity reduces cellular viability [15,16]. Moreover, studies indicate that LIDO promotes apoptosis through mitochondrial dysfunction [17]. AMX is a specialized cell culture medium. This in vitro study investigates the impact of AMX on lidocaine-induced toxicity in healthy colon cells.

## 2. Material and Method

### 2.1. Cell Culture

Ethical approval was received from our University’s Local Ethics Committee for Scientific Research, with decision number 08 taken at meeting 26 on 6 August 2021. The trial was conducted at our University’s Biochemistry Cell Culture Laboratory between 2021 and 2023.

With concerns surrounding lipid emulsions and a growing interest in alternative cytoprotective agents, we sought to evaluate the potential protective effects of AMX against lidocaine-induced cytotoxicity. Since the gastrointestinal tract is a major site of systemic drug metabolism and toxicity, we selected CCD-18Co (CRL-1459™, ATCC, Manassas, VA, USA), a healthy human colon cell line, as a representative in vitro model to study the cellular effects of lidocaine and to evaluate the protective role of AMX. These cells were cultured and expanded in EMEM medium enriched with 10% fetal bovine serum and 1% penicillin/streptomycin at 37 °C in 5% CO_2_.

Four groups were established for the study. **Control group:** This group consisted solely of CCD-18Co cells without the administration of any drugs as per the study design. **Lidocaine group (LIDO):** CCD-18Co cells were exposed to lidocaine at specified concentrations according to the study design. **AmnioMax^®^ group (AMX):** CCD-18Co cells were treated with AmnioMax^®^ at specified concentrations according to the study design. **Lidocaine + AmnioMax^®^ Group (LIDO + AMX):** This group was formed by treating CCD-18Co cells with lidocaine at concentrations determined in the study design, along with the maximum proliferative concentration of **AMX**. The half-maximal effective concentration (EC_50_) for **AMX** was calculated. The maximum proliferative concentration of **AMX** was determined as 60% to evaluate its protective efficacy, and cell viability assays were conducted on the LIDO + AMX group. LIDO (Sigma Aldrich, St. Louis, MO, USA; CAS number: 137-58-6) and AMX (Thermo Fisher, Waltham, MA, USA; CAS number: 11269016) used in the study were incubated at 37 °C for 24 h to examine their cell viability, genotoxicity, and apoptosis. Each concentration was tested in quadruplicate in the study. The concentrations of agents used in the study were 0.06–0.13–0.25–0.5–1–2–3–4–5–10% for lidocaine and 0.625–1.25–2.5–5–10–20–40–60–80–100% for AmnioMax^®^.

### 2.2. Cytotoxicity Assay

A luminometric ATP assay (CellTiter-Glo^®^ Luminescent Assay kit, Promega Corporation, Madison, WI, USA) was used to detect cell viability in CCD-18Co cells. It is a homogeneous method that measures the amount of ATP, serving as an indicator of viable cells. In the presence of intracellular ATP, luciferin in the medium is converted to oxyluciferin by the action of recombinant luciferase enzyme. Luminescence is emitted [18].

A total of 7.5 × 10^3^ cells were seeded into each well of opaque white 96-well plates. The cells were divided into four equal groups. The control group did not receive any treatment. The LIDO group ranged from 0.06% to 10%, and the AMX group ranged from 0.625% to 100%, diluted in phosphate-buffered saline (PBS), followed by a 24-h incubation. Using non-linear regression analysis, the EC_50_ value for AMX was determined to be 60% from the concentration–response curves, and the LIDO + AMX group was treated at concentrations ranging from 0.06% to 10% and subjected to a 24-h incubation. Following the incubation period, Following the addition of ATP solution, luminescence was detected within 5 min using a multimode plate reader (BioTek, Synergy™ HTX Flash Multimode Reader, Shoreline, WA, USA) with a luminometric method. Luminescence emitted in the presence of ATP was reported as relative luminescence units (RLU). The viability of cells was compared to that of the control group, which was set at 100%. All concentrations in the groups were in quadruplicate.

### 2.3. Measurement of Intracellular Reactive Oxygen Species

The production of intracellular reactive oxygen species (iROS) was assessed using the fluorescent signal indicator H_2_DCF-DA (Thermo Fisher Scientific^®^, Hillsboro, OR, USA). H_2_DCF-DA, initially colorless, reacts with ROS in the environment and converts to green fluorescent dichlorofluorescein (DCF) [18]. There is a positive correlation between increased ROS levels and emitted fluorescence.

Twenty-four hours after seeding 7.5 × 10^3^ cells per well into black opaque 96-well plates, the LIDO group received lidocaine at concentrations ranging from 0.06% to 10%, while the LIDO + AMX group was treated with the EC_50_ for AMX set at 60%. Following incubation and aspiration, the wells were washed three times with 1× dPBS. Next, 10 μM H_2_DCF-DA was added and incubated at 37 °C for 30 min. The fluorescence intensity of the resulting DCF was measured at Ex:488 nm/Em:525 nm using a multimode plate reader (BioTek, Synergy™ HTX Flash Multimode Reader, Shoreline, WA, USA). ATP levels were quantified and analyzed in relation to the control group, which was treated with 0.1% DMSO for both ROS and ATP measurements.

### 2.4. Measurement of Intracellular Glutathione

A luminometric glutathione (GSH) assay (GSH-Glo™ Glutathione Assay kit, Promega Corporation, Fitchburg, WI, USA) was employed to measure intracellular GSH levels, which utilizes the enzyme glutathione-S-transferase to reduce glutathione and convert the luciferin-NT substrate into luciferin. During this conversion, ATP is produced. The generated luciferin is then converted to oxyluciferin by recombinant luciferase enzyme, emitting luminescence [19].

Twenty-four hours after seeding 7.5 × 10^3^ cells per well into white opaque 96-well plates, the LIDO group was treated ranging from 0.06% to 10%, while the LIDO + AMX group was treated with the EC_50_ for AMX determined as 60%. After the addition of the GSH solution, luminescent signals were captured within 5 min using a multimode plate reader (BioTek, Synergy™ HTX Flash Multimode Reader, Shoreline, WA, USA). The results were calculated relative to ATP levels and compared with the control group, which was treated with 0.1% DMSO (GSH/ATP).

### 2.5. Measurement of DNA Damage

Genotoxic or DNA damage was assessed using the alkaline single-cell gel electrophoresis technique (Comet Assay), as described by Singh et al. [20]. This technique relies on the varying migration patterns of DNA fragments in an electric field, influenced by their size and charge. Following this principle, cells were embedded in agarose and then lysed to release their DNA. If the DNA is undamaged, it remains compact and does not form a tail. However, when DNA is damaged and fragmented, it migrates away from the nucleus during electrophoresis, forming a characteristic tail-like structure. These fragments appear as tails on ethidium bromide staining, an indication of DNA damage [20,21].

To determine the genotoxic potential of LIDO and AMX, 50 × 10^3^ cells per well were seeded into six-well plates, and the cells were treated with LIDO at concentrations ranging from 0.06% to 10% in the LIDO group, and the LIDO + AMX group was treated with the EC_50_ for AMX set at 60%. After 24 h of treatment, the cells were detached using trypsin-EDTA. Cells were washed with 1× PBS and centrifuged at 500× *g* for 5 min at +4 °C. After discarding the supernatant, 10 µL of the resulting cell suspension was mixed with 85 µL of 0.65% low melting point agarose (LMA) and applied to slides precoated with 1% normal melting point agarose (NMA). After the agarose solidified, the slides were incubated in lysis solution at +4 °C for a minimum of 4 h. Thereafter, the samples were rinsed with 1× PBS and incubated in electrophoresis buffer at +4 °C for 40 min to allow for DNA unwinding. Electrophoresis was subsequently conducted at +4 °C for 25 min using a voltage of 26 V and a current of 300 mA to allow the migration of fragmented DNA. Following electrophoresis, the slides were gently washed three times with neutralization buffer to remove excess alkali and restore a neutral pH. The DNA was then fixed by immersing the slides in ethanol to preserve the gel structure and prevent further diffusion. After air-drying at room temperature, the samples were stained with ethidium bromide at a final concentration of 2 μg/mL. The stained slides were visualized under a fluorescence microscope to assess DNA damage, with comet tail formation indicating the presence and extent of strand breaks (Nikon Eclipse Ts2, Tokyo, Japan). DNA damage was quantified by analyzing the percentage of DNA in the comet tails using Comet Assay IV analysis software 4.2 (Instem Group of Companies, Stone, UK).

### 2.6. Measurement of Apoptosis

The morphological changes in cells were assessed by acridine orange/ethidium bromide (AO/EB) staining. AO penetrates and stains both live and dead cells, emitting green fluorescence, whereas EB only enters cells with compromised membrane integrity, staining them red. Healthy cells appear uniformly green, early apoptotic cells show bright green dots, late apoptotic cells appear orange, and necrotic cells appear as irregularly shaped red cells [22]. Cells seeded at 50 × 10^3^ per well in 6-well plates were treated with LIDO at concentrations ranging from 0.06% to 10% in the LIDO group, and with a combination of LIDO + AMX group, with the EC_50_ for AMX set at 60%. Twenty-four hours after treatment, the cells were detached using trypsin-EDTA and centrifuged at 500× *g* for five minutes at +4 °C, followed by discarding the supernatant. Approximately equal volumes of the cell suspension and the dye solution were gently mixed to ensure even staining. The mixture was added onto a clean slide, and the slide was cover-slipped. The images of a minimum of 120 cells were assessed using a fluorescence microscope (Nikon Eclipse Ts2, Tokyo, Japan).

### 2.7. Measurement of Intracellular Calcium

Intracellular calcium (Ca) levels were assessed using the fluorescent dye Fura^2^AM [23]. Cells seeded at 7.5 × 10^3^ per well in black opaque 96-well plates were treated with LIDO at concentrations ranging from 0.06% to 10% in the LIDO group, and with a combination of LIDO and AMX in the LIDO + AMX group, with the EC_50_ for AMX set at 60%. After incubation, the media were aspirated, and cells were washed with 1× HBSS. A total of 100 µL of 5 µM Fura^2^AM was added to each well and incubated at room temperature for 30 min. After washing the cells twice with 1× HBSS, they were incubated with 1× HBSS for 45 min at room temperature. The fluorescence intensity was measured using a fluorescence plate reader (BioTek, Synergy™ HTX Flash Multi-Mode Reader, Shoreline, WA, USA) at Ex:340 nm/Em:380 nm. The results were expressed as calcium/ATP ratios and evaluated relative to the control group, which received 0.1% DMSO.

### 2.8. Measurement of Mitochondrial Membrane Potential

An important parameter of mitochondrial function and an indicator of cellular health is mitochondrial membrane potential (MMP). A decrease in MMP signifies loss of mitochondrial membrane integrity and initiation of pro-apoptotic signaling [24]. In this protocol, we used the 3,3′-dihexyloxacarbocyanine iodide (DiOC_6_(3)), a cell-permeant, green-fluorescent, lipophilic dye accumulating in mitochondria.

Cells seeded at 15 × 10^3^ per well in black opaque 96-well plates were, after 24 h, treated with lidocaine at concentrations ranging from 0.06% to 10% in the LIDO group, and with a combination of LIDO and AMX in the LIDO + AMX group, with the EC_50_ for AMX set at 60%. No treatment was applied to the control group. After 24 h of incubation, the cells were detached. Cells were incubated with 40 nM DiOC6(3) for 15 min at 37 °C to assess mitochondrial membrane potential. Fluorescence intensity was then measured at an excitation wavelength of 484 nm and an emission wavelength of 501 nm using a fluorescence plate reader (BioTek, Synergy™ HTX Flash Multimode Reader, Shoreline, WA, USA). The results were expressed as MMP/ATP ratios and compared to the control group.

### 2.9. Statistical Analysis

The SPSS software package (Version 25, Chicago, IL, USA) for Windows was used for all statistical analyses. Given the fact that we used cell culture in our study, the sample size was determined based on a post hoc power analysis using G*Power, which confirmed that the study had sufficient power to detect significant differences among groups. Each experimental condition was tested with at least four replicates. The EC_50_ concentrations of the drugs for AMX on healthy colon cells were determined using nonlinear regression analysis. The results were expressed as mean ± standard deviation, and statistical significance was determined using one-way ANOVA for data from all experiments. The correlations between all measured parameters were analyzed using the Pearson correlation coefficient, with a *p*-value of less than 0.05 considered statistically significant.

## 3. Results

**Figure 1:**
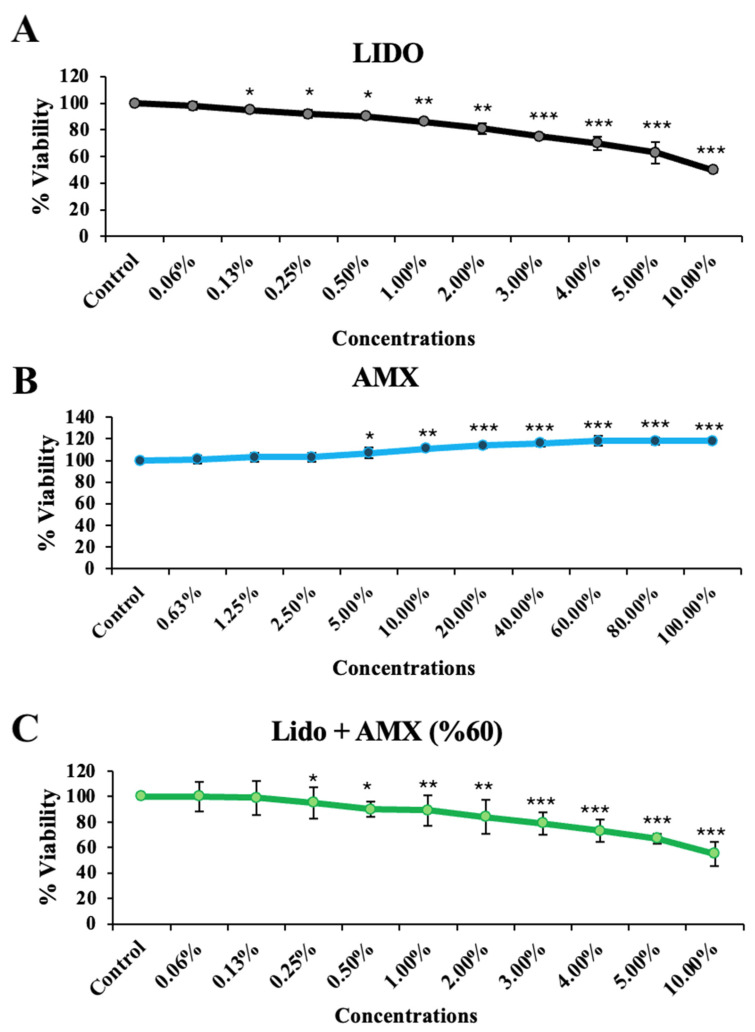
The effects of increasing concentrations of (**A**) lidocaine (LIDO), (**B**) AmnioMax^®^ (AMX), and (**C**) combined treatment (LIDO + AMX) on the viability of healthy colon CCD-18Co cells. Statistical significance: *p <* 0.05, * = *p* < 0.05, ** = *p* < 0.01, *** = *p* < 0.001. All experimental data are presented as the mean ± standard deviation (SD), calculated from four independent experiments conducted under the same conditions.

This study investigating the effects of AMX on LIDO-induced toxicity in healthy colon cell culture found that LIDO, AMX, and their combination LIDO + AMX each impacted viability in CCD-18Co cells (Figure 1). Exposure to increasing concentrations of LIDO significantly reduced cellular viability in CCD-18Co cells compared to the control group. For CCD-18Co cells, a significant reduction in viability was observed at a LIDO concentration of 0.13% (*p <* 0.05). The viability continued to decrease with increasing LIDO concentrations (*p <* 0.05 for 0.13–0.5%, *p <* 0.01 for 1–2%, *p <* 0.001 for 3–10%).

Conversely, AMX significantly increased cellular viability in CCD-18Co cells compared to the control group (*p* < 0.001). A significant increase was observed at 5% AMX (*p <* 0.05), with the maximum proliferative effect at 60% (*p <* 0.001). No statistically significant difference was found for concentrations above 60%, and similar effects on cellular viability were observed (*p <* 0.001 for 60–80–100%).

In the LIDO + AMX group, viability was significantly lower than in the control group at all tested concentrations (*p <* 0.001). However, cytotoxicity was significantly reduced compared to the LIDO-only group (*p <* 0.05). The protective effect of AMX became more pronounced at LIDO concentrations above 2% (*p* < 0.05).

When combined, AMX demonstrated a cytoprotective effect against LIDO-induced cytotoxicity, as evidenced by the statistically significant differences between the LIDO and LIDO + AMX groups at concentrations above 2% (*p <* 0.05), see Figure 1.

**Figure 2:**
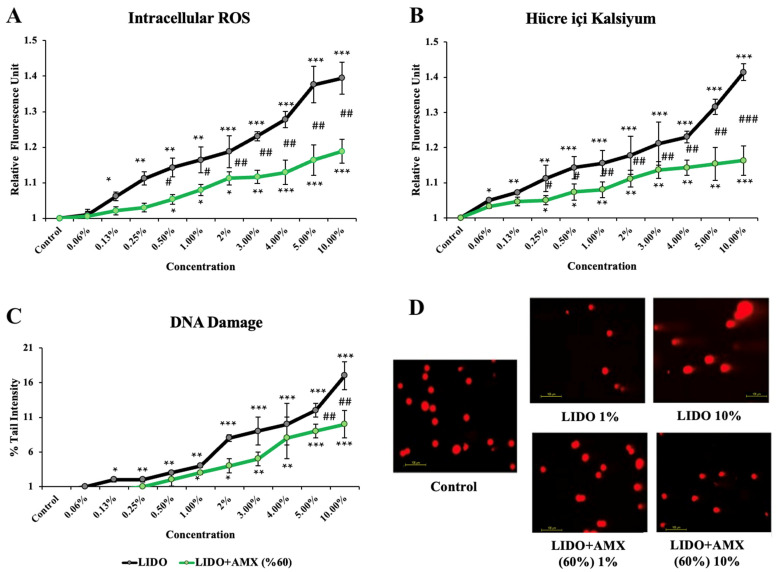
The effects of lidocaine (LIDO) and the combination of LIDO with 60% AmnioMax^®^ (AMX) on (**A**) intracellular ROS, (**B**) intracellular calcium levels, (**C**) DNA damage in CCD-18Co cells, and (**D**) fluorescence microscopy images (scale bar: 100 μm) of DNA damage levels in CCD-18Co cells treated with LIDO (1–10%) and the combination of LIDO with AMX (60%). Differences in CCD-18Co cells: * *p* < 0.05; ** *p <* 0.01; *** *p <* 0.001, differences between both groups are considered statistically significant at # *p <* 0.05; ## *p <* 0.01; ### *p <* 0.001. Data are represented as mean ± SD of four independent experiments.

The effects of lidocaine and the combination of LIDO with 60% AMX on iROS, intracellular Ca levels, and DNA damage in cells compared to the control group are shown in Figure 2.

LIDO treatment led to a significant increase in iROS levels compared to the control group (*p <* 0.05 for 0.13%, *p <* 0.01 for 0.25–1%, *p <* 0.001 for 2–10%). In the LIDO + AMX (60%) group, increasing concentrations of LIDO also resulted in higher iROS levels compared to the control group without treatment (*p <* 0.05 for 0.5–2%, *p <* 0.01 for 3%, *p <* 0.001 for 4–10%). However, when comparing LIDO and LIDO + AMX groups, statistically significant differences were observed at concentrations between 0.5% and 10% with the LIDO + AMX combination exhibiting reduced iROS formation (*p <* 0.05 # for 0.5–1%, *p <* 0.01 ## for 2–10%).

Increasing concentrations of LIDO-induced intracellular Ca levels were compared to the control group (*p <* 0.05 for 0.06%, *p <* 0.01 for 0.13–0.25%, *p <* 0.001 for 0.5–10%). In the LIDO + AMX group, LIDO exposure also resulted in increased intracellular Ca levels (*p <* 0.05 for 0.25–0.5%, *p <* 0.01 for 1–5%, *p <* 0.001 for 10%) compared to the control group. Notably, a significant difference in intracellular Ca levels was found between the LIDO and LIDO + AMX groups across concentrations of 0.25% to 10%. AMX (60%) mitigated the rise (*p <* 0.05 # for 0.25–0.5%, *p <* 0.01 ## for 1–5%, *p <* 0.001 ### for 10%).

Compared to the control, LIDO administration significantly increased DNA damage levels (*p <* 0.05 for 0.13%, *p <* 0.01 for 0.25–1%, *p <* 0.001 for 2–10%). A similar trend was observed in the LIDO + AMX group, where higher LIDO concentrations induced higher DNA damage (*p <* 0.05 for 1–2%, *p <* 0.01 for 3–4%, *p <* 0.001 for 5–10%). However, when comparing DNA damage between LIDO and LIDO + AMX groups, significant reductions were observed at 5% and 10% in the LIDO + AMX group (*p <* 0.01 ## for 5% and 10%), indicating a protective effect of AMX.

**Figure 3:**
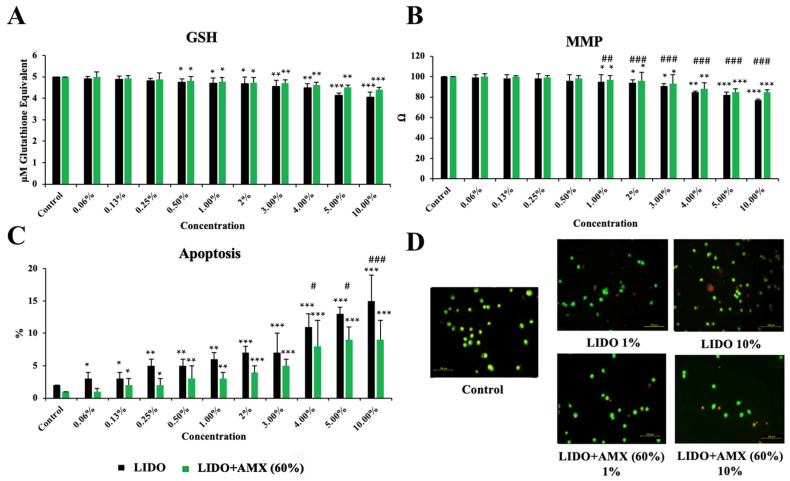
The effects of lidocaine (LIDO) and the combination of LIDO with 60% AmnioMax^®^ (LIDO + AMX) on intracellular (**A**) GSH, (**B**) mitochondrial membrane potential, (**C**) apoptosis in healthy colon cells, and (**D**) fluorescence microscopy images (scale bar: 100 μm) of apoptosis levels in CCD-18Co cells treated with lidocaine (1–10%) and the combination of lidocaine with AMX (60%). Differences in CCD-18Co cells: * *p <* 0.05; ** *p <* 0.01; *** *p <* 0.001; differences between both groups are considered statistically significant at # *p <* 0.05; ## *p <* 0.01; ### *p <* 0.001. Data are represented as mean ± SD of four independent experiments.

Figure 3 shows the effects of LIDO and the LIDO + AMX 60% combination on GSH, MMP, and apoptosis levels in CCD-18Co cells, compared to the control group.

GSH levels exhibited a concentration-dependent decline in the LIDO group relative to the control (*p <* 0.05 for 0.5–2%, *p <* 0.01 for 3–4%, *p <* 0.001 for 5–10%). Similarly, in the LIDO + AMX group, increasing concentrations of lidocaine led to a significant reduction in GSH levels (*p <* 0.05 for 0.5–2%, *p <* 0.01 for 3–5%, *p <* 0.001 for 10%). However, no statistically significant difference was found between the LIDO and LIDO + AMX groups regarding GSH levels.

MMP levels also decreased progressively with higher LIDO group when compared to the control group (*p <* 0.05 for 1–3%, *p <* 0.01 for 4%, *p <* 0.001 for 5–10%). A similar pattern was detected in the LIDO + AMX group, where MMP levels dropped in response to increasing LIDO concentrations (*p <* 0.05 for 1–3%, *p <* 0.01 for 4%, *p <* 0.001 for 5–10%). When comparing the LIDO and LIDO + AMX groups, significant differences emerged across concentrations ranging from 1% to 10%, with MMP levels remaining significantly higher in the LIDO + AMX group (*p <* 0.01 ## for 1% and *p <* 0.001 ### for 2–10%).

Apoptosis levels were markedly elevated in the LIDO group compared to the control group (*p <* 0.05 for 0.06–0.13%, *p <* 0.01 for 0.25–1%, *p <* 0.001 for 2–10%). A comparable increase in apoptosis was observed in the LIDO + AMX group following LIDO exposure (*p <* 0.05 for 0.13–0.25%, *p <* 0.01 for 0.5–1%, *p <* 0.001 for 2–10%). Statistically significant differences in apoptosis levels between the LIDO and LIDO + AMX groups were detected at concentrations of 4–5% and 10%. Additionally, cellular viability was significantly higher in the LIDO + AMX group compared to the LIDO group, highlighting the protective effect of 60% AMX against LIDO-induced cytotoxicity (*p <* 0.05 # for 4–5% and *p <* 0.01 ## for 10%).

## 4. Discussion

In this study, we evaluated the efficacy of AMX in protecting healthy colon cell cultures from LIDO-induced toxicity. Our findings demonstrated a concentration-dependent decrease in cellular viability with increasing LIDO exposure, whereas higher concentrations of AMX significantly increased enhanced viability. Additionally, the combination of AMX with LIDO resulted in notably lower cytotoxicity compared to LIDO alone, suggesting a protective effect. The presence of AMX helped preserved cell viability and mitigated LIDO-induced cytotoxicity. Furthermore, our analysis revealed a negative correlation between cellular viability and iROS, Ca, DNA damage, and apoptosis, while intracellular GSH and MMP levels showed a positive correlation.

A review of 291 cases investigating myotoxicity associated with local anaesthetics reported that LIDO, bupivacaine, and mepivacaine induce apoptosis and cell death by causing mitochondrial damage and increasing intracellular Ca levels at both low and high concentrations [25]. It has been found that damage occurs more rapidly and extensively at high concentrations. In a cell culture study conducted by Park et al. [26], the neurotoxic effects of LIDO, bupivacaine, procaine, mepivacaine, ropivacaine, and levobupivacaine were examined in Schwann cells. Interestingly, only bupivacaine exposure led to a significant reduction in cellular viability, whereas LIDO did not exhibit a similar effect in that study. Further experiments revealed that bupivacaine-induced apoptosis was linked to increased ROS production. The discrepancy between these findings and those of this study may be attributed to the lower LIDO concentration used (1 mM) in Park et al.’s research. One of the key features of this study was the wide range of LIDO concentrations that were applied, which enhances the reliability of our results. We observed that LIDO exposure led to increased iROS levels and Ca concentrations while decreasing MMP levels in healthy colon epithelial cells. These findings indicate a strong association between LIDO-induced toxicity and oxidative stress, calcium dysregulation, and mitochondrial dysfunction.

The beneficial effects of AMX have been first demonstrated in our in vitro study on cell cultures. Previous experimental studies have shown that AMX exerts positive effects in reversing ischemia/reperfusion injury [27]. Additionally, AMX has been reported to reduce both necrosis and cell death in an experimental dorsal skin flap model [28]. Similarly, when compared with platelet-rich plasma for treating tracheal injury, cell cultures derived from amniotic fluid have exhibited greater benefits in promoting wound healing [29]. According to these findings, the AMX-treated group experienced lower oxidative stress and showed more favorable outcomes in connective tissue regeneration and new blood vessel formation. This study provides novel insights into the protective effects of AMX against LIDO-induced cytotoxicity in CCD-18Co. Consistent with previous findings, we observed a dose-dependent reduction in cellular viability following LIDO exposure, suggesting its cytotoxic effects at increasing concentrations. Notably, our results align with studies indicating that LIDO induces apoptosis through mitochondrial dysfunction, ultimately compromising cell survival.

### Study Limitations

This study lacked in vivo experiments, which are essential for confirming the protective effects of AMX against lidocaine toxicity in a whole organism. While in vitro findings provide valuable insights, animal models would offer a more comprehensive understanding by better replicating physiological conditions, including pharmacokinetics, systemic interactions, and potential therapeutic outcomes. Future studies should incorporate in vivo approaches to validate these findings and assess the clinical relevance of AMX in mitigating lidocaine-induced toxicity.

## Data Availability

The original contributions presented in this study are included in the article. Further inquiries can be directed to the corresponding author.

## References

[1] Wadlund D.L. (2017). Local anaesthetic systemic toxicity. AORN J..

[2] Yang S., Abrahams M.S., Hurn P.D., Grafe M.R., Kirsch J.R. (2011). Local anesthetic Schwann cell toxicity is time and concentration dependent. Reg. Anesth. Pain Med..

[3] Yasmeen S., Liao X., Khan F.U., Ihsan A.U., Li X., Li C., Chen D., Yu F., Wang Z., Sembatya K.R. (2019). A novel approach to devise the therapy for ventricular fibrillation by epicardial delivery of lidocaine using active hydraulic ventricular attaching support system: An experimental study in rats. J. Biomed. Mater. Res. Part B Appl. Biomater..

[4] Mirshahidi S., Shields T.G., de Necochea-Campion R., Yuan X., Janjua A.B., Williams N.L., Mirshahidi H.R., Reeves M.E., Duerksen-Hughes P., Zuckerman L.M. (2021). Bupivacaine and Lidocaine Induce Apoptosis in Osteosarcoma Tumor Cells. Clin. Orthop. Relat. Res..

[5] Neal J.M., Barrington M.J., Fettiplace M.R., Gitman M., Memtsoudis S.G., Mörwald E.E., Rubin D.S., Weinberg G. (2018). The Third American Society of Regional Anesthesia and Pain Medicine Practice Advisory on Local Anesthetic Systemic Toxicity: Executive Summary 2017. Reg. Anesth. Pain Med..

[6] American College of Medical Toxicology (2017). ACMT Position Statement: Hospital Privileges for Physicians Practicing Medical Toxicology. J. Med. Toxicol..

[7] Van den Berg M.J., Bosch F.H. (2016). Case report: Hemodynamic Instability Following Severe Metoprolol and Imipramine Intoxication Successfully Treated with Intravenous Fat Emulsion. Am. J. Ther..

[8] Chhabria B.A., Bhalla A., Shafiq N., Kumar S., Dhibar D.P., Sharma N. (2019). Lipid emulsion for acute organophosphate insecticide poisoning-a pilot observational safety study. Clin. Toxicol..

[9] Liu Y., Zhang J., Yu P., Niu J., Yu S. (2021). Mechanisms and Efficacy of Intravenous Lipid Emulsion Treatment for Systemic Toxicity From Local Anesthetics. Front. Med..

[10] Hoshino R., Kamiya Y., Fujii Y., Tsubokawa T. (2017). Lipid emulsion injection-induced reversal of cardiac toxicity and acceleration of emergence from general anaesthesia after scalp infiltration of a local anaesthetic: A case report. JA Clin. Rep..

[11] Hayes B.D., Gosselin S., Calello D.P., Nacca N., Rollins C.J., Abourbih D., Morris M., Nesbitt-Miller A., Morais J.A., Lavergne V. (2016). Systematic review of clinical adverse events reported after acute intravenous lipid emulsion administration. Clin. Toxicol..

[12] Fairbairn N.G., Randolph M.A., Redmond R.W. (2014). The clinical applications of human amnion in plastic surgery. J. Plast. Reconstr. Aesthetic Surg..

[13] Klemmt P.A., Vafaizadeh V., Groner B. (2011). The potential of amniotic fluid stem cells for cellular therapy and tissue engineering. Expert Opin. Biol. Ther..

[14] Shamsnajafabadi H., Soheili Z.S. (2022). Amniotic fluid characteristics and its application in stem cell therapy: A review. Int. J. Reprod. Biomed..

[15] Seshadri V., Coyle C.H., Chu C.R. (2009). Lidocaine Potentiates the Chondrotoxicity of Methylprednisolone. Arthrosc. J. Arthrosc. Relat. Surg..

[16] Karpie J.C., Chu C.R. (2007). Lidocaine Exhibits Dose- and Time-Dependent Cytotoxic Effects on Bovine Articular Chondrocytes in Vitro. Am. J. Sports Med..

[17] Werdehausen R., Braun S., Fazeli S., Hermanns H., Hollmann M.W., Bauer I., Stevens M.F. (2012). Lipophilicity but not stereospecificity is a major determinant of local anaesthetic-induced cytotoxicity in human T-lymphoma cells. Eur. J. Anaesthesiol..

[18] Kocyigit A., Guler E.M., Karatas E., Caglar H., Bulut H. (2018). Dose-dependent proliferative and cytotoxic effects of melatonin on human epidermoid carcinoma and normal skin fibroblast cells. Mutat. Res. Genet. Toxicol. Environ. Mutagen..

[19] Günes-Bayir A., Kiziltan H.S., Kocyigit A., Güler E.M., Karataş E., Toprak A. (2017). Effects of natural phenolic compound carvacrol on the human gastric adenocarcinoma (AGS) cells in vitro. Anti-Cancer Drugs.

[20] Singh N.P., Danner D.B., Tice R.R., Brant L., Schneider E.L. (1990). DNA damage and repair with age in individual human lymphocytes. Mutat. Res..

[21] Demirbag R., Yilmaz R., Gur M., Kocyigit A., Celik H., Guzel S., Selek S. (2005). Lymphocyte DNA damage in patients with acute coronary syndrome and its relationship with severity of acute coronary syndrome. Mutat. Res..

[22] Kasibhatla S., Amarante-Mendes G.P., Finucane D., Brunner T., Bossy-Wetzel E., Green D.R. (2006). Acridine Orange/Ethidium Bromide (AO/EB) Staining to Detect Apoptosis. CSH Protoc..

[23] Untario N., Dewi T.C., Widodo M.A., Rahaju P. (2017). Effect of Tetrodotoxin from Crude Puffer Fish (Tetraodon fluviatilis) Liver Extract on Intracellular Calcium Level and Apoptosis of HeLa Cell Culture. J. Trop. Life Sci..

[24] Rottenberg H., Wu S. (1998). Quantitative assay by flow cytometry of the mitochondrial membrane potential in intact cells. Biochim. Biophys. Acta (BBA) Mol. Cell Res..

[25] Hussain N., McCartney C.J.L., Neal J.M., Chippor J., Banfield L., Abdallah F.W. (2018). Local anaesthetic-induced myotoxicity in regional anaesthesia: A systematic review and empirical analysis. Br. J. Anaesth..

[26] Park C.J., Park S.A., Yoon T.G., Lee S.J., Yum K.W., Kim H.J. (2005). Bupivacaine Induces Apoptosis via ROS in the Schwann Cell Line. J. Dent. Res..

[27] Dundar T.T., Yildiz K., Tosuner Z., Mihrapoğlu S.L., Kitiş S. (2020). Mcfarlane rat dorsal cilt flep modelinde amniomax’in nekroz önleyici etkisinin araştırılması. Kocatepe Med. J..

[28] Mirapoglu S.L., Guler E.M., Tok O.E., Aydogdu I., Cay A., Camli M.F., Kocyigit A., Canter H.I., Yildiz K. (2021). Effects of Platelet Rich Plasma and Amniotic Cell Culture Medium on Wound Healing Following Experimental Animal Tracheal Injury Model: A Comparative Study. J. Craniofac. Surg..

[29] Aydogdu I., Karaca E., Coban G., Cay A., Guler E.M., Kocyigit A., Uzun E., Aydoğdu Y.E., Metin H., Miçooğullari U. (2021). An investigation of the effects of amniotic fluid on experimental ischemia/reperfusion damage in rat testes. J. Pediatr. Urol..

